# Implications of adopting the WHO 2006 Child Growth Standard in the UK: two prospective cohort studies

**DOI:** 10.1136/adc.2007.126854

**Published:** 2007-10-01

**Authors:** C Wright, R Lakshman, P Emmett, K K Ong

**Affiliations:** 1 Division of Developmental Medicine, University of Glasgow, Glasgow, UK; 2 MRC Epidemiology Unit, Cambridge, UK; 3 Community based Medicine, University of Bristol, Bristol, UK; 4 Department of Paediatrics, University of Cambridge, Cambridge, UK

## Abstract

**Background:**

The WHO 2006 Child Growth Standard is based on data from international optimally nourished breastfed infants from birth to age 5 years.

**Objective:**

To assess the potential effect of its use on weight and growth monitoring of UK children.

**Participants:**

Full-term members of two population-based UK birth cohorts: the Children in Focus sub-cohort of the Avon Longitudinal Study of Parents and Children (ALSPAC) (n = 1335) and the Gateshead Millennium Baby Study (GMS; n = 923).

**Design:**

Growth data from birth to 5 years were converted into z-scores relative to the WHO 2006 standard.

**Results:**

Compared with the WHO standard, both UK cohorts had higher birth weights (mean z-scores: GMS, 0.17; ALSPAC, 0.34) and ALSPAC had higher birth lengths. After birth, length showed a good fit at all ages. By 2–4 months, both cohorts were similar in weight to the WHO median (mean WHO weight z-score at 4 months: GMS, 0.01; ALSPAC, −0.07), but thereafter the UK cohorts were heavier (mean WHO weight z-score at 12 months: GMS, 0.57; ALSPAC, 0.65). At age 12 months, the risk of being classified as underweight (weight <2nd centile) was considerably lower according to the WHO standard than by the UK 1990 Growth Reference (RR = 0.15, 95% CI = 0.07 to 0.32), and the risk of being classified as obese at 4–5 years (body mass index >98th centile) was slightly increased (RR = 1.35, 95% CI = 1.02 to 1.78).

**Conclusions:**

Adoption of the WHO 2006 Growth Charts would set a markedly lower standard of weight gain beyond the age of 4 months for UK infants and could support efforts to avoid future childhood obesity. However, the WHO standard is not representative of size at birth in the UK.

The World Health Organisation (WHO) Child Growth Standard for infants and children up to the age of 5 years was published in April 2006. It is based on the growth of healthy breastfed children in optimal conditions between 1997 and 2003 from six different countries: Brazil, Ghana, India, Norway, Oman and USA.^1^ ^2^ The WHO Multicentre Growth Reference Study (MGRS) collected data for ∼8500 children who were exclusively breast fed for the first 4 months and were living in a well-supported health environment. In consequence, the WHO aims to provide for the first time a standard on “how children should grow”, rather than a traditional growth reference that describes “how children are growing”.

There is an understandable enthusiasm for the idea of adopting these charts in the UK, but before doing so it is important to assess how well UK children match, or diverge from, the new charts, in order to understand the implications for growth monitoring and clinical care. We have explored this question using data from two representative UK birth cohorts.

## METHODS

The two datasets used were from the Gateshead Millennium Baby Study (GMS) and the Children in Focus sub-sample of the Avon Longitudinal Study of Parents and Children (ALSPAC), which between them provide detailed growth data spanning the entire period of the new charts. GMS is a prospective population-based cohort study of feeding and growth in infancy comprising 1029 babies born between June 1999 and May 2000 in Gateshead, an urban borough in the North of England. For this analysis, data from 923 full-term infants were used.^3^ Birth weight was retrieved from the maternity record, and weights at 12 days, 6–8 weeks, 4 months and 12 months were obtained from the Personal Child Health Records as well as height and weight at school entry.^3^ Half were breast fed at birth, but only 10% continued breast feeding beyond 4 months.

The ALSPAC Children in Focus sub-cohort includes 1335 full-term infants born in Avon, south-west England, between June and December 1992. Weight and length/height measurements were collected at research clinics at birth, 4 months, 8 months, 12 months, 18 months, 24 months and 5 years.^4^ Just less than half (46%) were breast fed at age four months (including up to one formula feed per day).

For each child, age- and sex-adjusted z-scores for weight, length (height at >2 years old) and body mass index (BMI) were calculated using exact ages at measurement by comparison with both the WHO 2006 and the UK 1990 growth data using software provided respectively by the WHO and the Child Growth Foundation (London, UK). Conditional weight gain was calculated to account for regression to the mean.^3^ Poor infant weight gain was defined as a change in weight SD score <−1.33 SD, which is equivalent to downward crossing through two major centile lines on each growth chart.

Both studies received appropriate ethics committee approval and obtained informed written consent from each participant.

## RESULTS

### Comparisons with UK 1990

**Figure 1:**
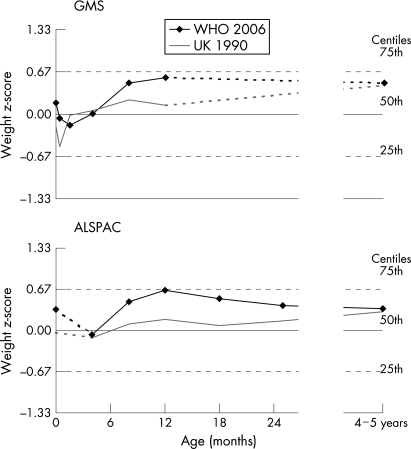
Mean z-scores for weight from birth to 24 months and at 4–5 years, according to WHO 2006 Growth Standard (WHO 2006) or the British 1990 Growth Reference (UK 1990) for the Gateshead Millennium Baby Study (GMS) and the Children in Focus sub-cohort of the Avon Longitudinal Study of Parents and Children (ALSPAC). Dotted lines in each panel indicate the time periods with less density of measurements.

Both cohorts showed a reasonably good fit with the UK 1990 reference during the first year of life, as indicated by mean weight and length z-scores close to zero (table 1 and fig 1). The only exception was a transient decline in weight z-score in GMS at age 12 days, which may be expected, as the UK 1990 reference makes no allowance for the physiological neonatal weight loss. By age 4–5 years, weight and BMI z-scores in both cohorts were higher than the UK 1990 average.

**Table 1:** z-Scores for length/height, weight and body mass index (BMI) from birth according to the WHO 2006 Growth Standard (WHO) or the British 1990 Growth Reference (UK1990) in the ALSPAC and GMS cohorts

Age	Numbers		Length/height SDS				Weight SDS				BMI SDS			
			WHO		UK1990		WHO		UK1990		WHO		UK1990	
	ALSPAC	GMS	ALSPAC	GMS	ALSPAC	GMS	ALSPAC	GMS	ALSPAC	GMS	ALSPAC	GMS	ALSPAC	GMS
Birth	1335	923	0.65 (1.04)	–	0.04 (1.00)	–	0.34 (1.01)	0.17 (1.07)	−0.03 (1.04)	−0.20 (1.09)	0.00 (0.98)	–	0.17 (1.00)	–
12 days	–	806	–	–	–	–	–	−0.07 (1.00)	–	−0.51 (1.03)	–	–	–	–
6–8 weeks	–	788	–	–	–	–	–	−0.17 (0.93)	–	−0.02 (0.99)	–	–	–	–
4 months	943	796	−0.03 (0.91)	–	−0.05 (0.90)	–	−0.07 (0.90)	0.01 (0.96)	−0.12 (0.97)	0.05 (1.03)	−0.07 (0.94)	–	−0.12 (1.29)	–
8 months	1231	601	0.15 (0.96)	–	0.08 (0.96)	–	0.46 (0.97)	0.49 (0.94)	0.10 (1.08)	0.22 (1.04)	0.50 (0.96)	–	0.09 (1.06)	–
1 year	1164	774	0.09 (0.95)	–	0.09 (0.94)	–	0.65 (0.93)	0.57 (0.94)	0.18 (1.05)	0.14 (1.05)	0.81 (0.89)	–	0.19 (1.01)	–
1.5 years	1088	–	−0.09 (0.97)	–	0.02 (0.96)	–	0.51 (0.91)	–	0.08 (1.04)	–	0.81 (0.89)	–	0.06 (1.03)	–
2 years	977	–	−0.14 (0.93)	–	−0.11 (0.93)	–	0.40 (0.92)	–	0.15 (1.04)	–	0.66 (0.93)	–	0.26 (1.03)	–
4–5 years	963	395*	−0.11 (0.91)	−0.16 (0.93)	0.08 (0.94)	−0.01 (0.97)	0.35 (0.90)	0.49 (0.96)	0.31 (1.00)	0.45 (1.03)	0.63 (0.92)	0.87 (0.99)	0.42 (0.99)	0.66 (0.99)

Values are mean (SD).

*281 GMS children aged >5 years at school entry measurement were excluded as they could not be compared with WHO.

ALSPAC, Children in Focus sub-cohort of Avon Longitudinal Study of Parents and Children; GMS, Gateshead Millennium Baby Study.

### Comparisons with WHO 2006

UK children had relatively high mean z-scores for birth weight and birth length compared with the WHO 2006 standard (table 1). After birth, z-scores for weight in the GMS children rapidly declined towards the WHO median by age 2 weeks, and in both cohorts weight showed a good fit up to 4 months (fig 1). Length and height in both cohorts showed a good fit at all ages after birth (table 1).

Between 4 months and 1 year, compared with the WHO standard, both cohorts showed a rapid rise in mean weight z-scores. After 1 year, the mean z-scores as assessed by the different growth reference data started to converge (table 1, fig 1).

By the WHO 2006 standard, infants were considerably less likely to be classified as underweight (weight <2nd centile; relative risk at 1 year = 0.15; 95% CI = 0.07 to 0.32) or having poor weight gain (downward-crossing through weight centiles) over the first year, compared with the UK 1990 Reference (table 2). Conversely the proportion of children classified as obese (BMI >98th centile) at age 4–5 years was slightly higher according to the WHO 2006 standard (relative risk = 1.35; 95% CI = 1.02 to 1.78; table 2).

**Table 2:** Percentages of children classified as underweight, poor infant weight gain, or obese according to the WHO Growth Standard (WHO) and the British 1990 Growth Reference (UK1990)

	ALSPAC		GMS		Combined:RR (95% CI)
	WHO	UK1990	WHO	UK1990	
Underweight					
6–8 weeks	–	–	3.6	2.9	1.12 (0.87 to 1.43)
4 months	2.0	2.4	2.1	2.4	0.86 (0.55 to 1.33)
8 months	0.7	2.6	0.7	1.8	0.30 (0.17 to 0.56)
1 year	0.3	2.4	0.4	2.5	0.15 (0.07 to 0.32)
1.5 years	0.6	2.9	–	–	0.22 (0.10 to 0.48)
Poor infant weight gain					
Birth to 1 year	1.7	7.1	1.6	5.4	0.24 (0.16 to 0.36)
6–8 weeks to 1 year	–	–	0.4	5.4	0.08 (0.03 to 0.24)
Obese (%)					
1 year	8.7	2.7	–	–	3.26 (2.21 to 4.83)
1.5 years	8.0	2.6	–	–	3.11 (2.06 to 4.71)
2 years	7.5	4.3	–	–	1.74 (1.20 to 2.51)
4–5 years	7.2	5.0	10.1	8.4	1.35 (1.02 to 1.78)

ALSPAC, Children in Focus sub-cohort of Avon Longitudinal Study of Parents and Children; GMS, Gateshead Millennium Baby Study; RR, relative risk for each outcome using the WHO standard, compared with the UK 1990 reference; Underweight, weight <2nd centile; Poor infant weight gain, conditional weight gain <−1.33 SD, equivalent to downward crossing through two major centile lines on each growth chart; Obese, body mass index >98th centile.

## DISCUSSION

In summary, adoption of the new WHO growth charts for UK children up to age 5 years would have a significant impact on the interpretation of their weight gain and growth. However, the effects are complex and appear to differ at various ages. The marked reduction in numbers of infants who would be classified as underweight or growth faltering beyond age 4 months is an expected consequence of the WHO’s decision to have the breastfed child as the normative model. However, UK infants would also be classified as being larger at birth, but not at 2–4 months, and would result in a complex pattern of weight centile changes over the first year for the average UK child (fig 1).

This analysis is based on data from two large representative UK birth cohorts, which between them allow comparison with the WHO charts at a wide range of ages. GMS provides detailed weight data early in infancy, and the ALSPAC provides both weight and height/length from infancy through to the pre-school years. At times of overlap, the two cohorts showed very close similarity in weights and heights, and, at least in infancy, they are also broadly similar to the UK 1990 reference. The gradual increase in weight z-scores by 4–5 years of age compared with the UK 1990 has been previously reported in ALSPAC and probably reflects the secular changes in UK children.^5^ We are therefore confident that our findings in these two cohorts may be extrapolated to contemporary UK children.

The WHO 2006 Child Growth Standard embodies a number of novel and admirable principles, with the aim of promoting optimal infant and childhood growth. Firstly, the international MGRS source data indicated for the first time that population differences in growth are avoidable, given optimum nutrition and living conditions.^6^ Secondly, the WHO has clearly placed the breastfed child as the norm for growth and development. Conditions of inclusion in the longitudinal component of the MGRS analysis were exclusive or predominant breast feeding up to age 4 months and partial breast feeding to at least 12 months. In consequence, the WHO feels able to publish a standard for optimal growth, rather than simply a description of current prevailing growth norms (a “reference”), which may not reflect ideal growth patterns.

**What is already known on this topic**

- The WHO published new growth charts in April 2006 based on infants of non-smoking, breastfeeding mothers living in optimal conditions in six countries.
- The WHO proposed that these set a standard for normal growth in infancy applicable throughout the world.

**What this study adds**

- At birth, UK children are longer and heavier than the WHO standard,
- After birth, the length of UK children matches the WHO standard closely.
- Use of the WHO standard would lead to far fewer UK children being classified as underweight or weight faltering in the first year, but more would be classified as overweight in the pre-school years.
- The WHO 2006 Growth Charts would set a lower standard of weight gain for UK infants.

However, our findings, particularly during the first 2 months, suggest that these standards may not be simply transferable to the UK. On the WHO chart, UK infants would appear larger than average at birth and then cross approximately half a centile space downwards in the first few weeks of life. The explanation for this may be that, although postnatal nutrition in the WHO MGRS cohort was optimal, intrauterine growth appeared to have been constrained, as size at birth was generally smaller than in the UK. In the MGRS constituent datasets, whereas mean birth weights in Norway and USA (3.5–3.6 kg) were similar to that in the UK, the populations from several other countries showed markedly lower mean birth weights (3.1 kg in India, and 3.2 kg in Oman), and this appears to correlate with differences in maternal size.^7^

The UK 1990 and other existing national growth charts do not allow for the rapid weight loss and recovery that normally occurs in the first 2 weeks of life.^8^ This is reflected in fig 1 by a transient dip in the GMS cohort UK 1990 weight SD scores at age 12 days, which probably corrected itself well before their next measurement at age 6 weeks. In contrast, the WHO standard does allow for normal neonatal weight loss.^2^ Therefore, the apparent downward shift in weight centile of UK children on the WHO chart after birth (fig 1) is not simply a transient physiological weight loss, but rather suggests that individual babies with low birth weight in the international MGRS birth cohort showed rapid catch-up growth after birth, even within the first 2 weeks.

Beyond the first 2–4 months, use of the WHO standard would make it much less likely for UK children to be classified as underweight or growth faltering. Recent work has revealed that mild degrees of weight faltering are unlikely to be associated with major social or medical disorders,^3^ and concerns have been expressed that unnecessary parental anxiety may be caused by over-diagnosis.^9^ A change to a new standard, with a more stringent and thus more specific lower threshold, may therefore be timely.

In contrast with underweight, adoption of the WHO growth chart would make UK infants and toddlers more likely to be classified as overweight or obese. There is a growing body of evidence that a higher plane of growth during infancy is associated with increased risk of obesity in children and adults.^10^ ^11^ Although it is not at all clear whether intervention in infancy can have a useful impact on later obesity, presenting the model of slower weight gain during later infancy prescribed by the WHO standard may be beneficial to the long-term health of these children.

The birth weight section of the WHO chart presents other difficulties, as there is no preterm element, which is a well-used feature of UK charts. These two issues taken together suggest that it may not be desirable for the UK to adopt the birth weight section of the WHO chart, beginning its use instead after the first 2 weeks.

In conclusion, the WHO 2006 Growth Standard places the breastfed child as the norm for growth. Its use would greatly reduce the numbers of UK infants classified as underweight and support efforts to avoid excess weight gain in infancy. However, the WHO 2006 Growth Standard is not representative of size at birth in the UK. In view of the resulting complex weight centile changes in the first few weeks of life, the potential confusion about feeding that this might raise with mothers, and also the absence of a preterm element to the WHO charts, the Department of Health Scientific Advisory Committee on Nutrition and the Royal College of Paediatrics and Child Health have recently jointly recommended that the WHO 2006 Growth Standard is appropriate for use in the UK children, but only from age 2 weeks.^12^ For birth weight, the UK 1990 reference would continue. The consequences of these recommendations for monitoring of infant weight gain in the UK are likely to be widespread and will need careful and coordinated consideration.
